# Atypical fibrous histiocytoma of the skull: a case report of temporal bone involvement and comprehensive literature review

**DOI:** 10.3389/fonc.2025.1663588

**Published:** 2026-01-28

**Authors:** Qiang Dong, Jing Shi, Beiyan Tang, Hongyu Wang, Chengliang Miao, Yongqiang Ma, Lei Duan, Guoqiang Yuan, Yawen Pan

**Affiliations:** 1 Department of Neurosurgery, Second Hospital of Lanzhou University, Lanzhou, Gansu, China; 2 Department of Urology, Second Hospital of Lanzhou University, Lanzhou, Gansu, China; 3 The Second Medical College of Lanzhou University, Lanzhou, Gansu, China; 4 Department of Neurosurgery, No.1 Hospital Of Yumen City, Yumen, Gansu, China; 5 Key Laboratory of Neurology of Gansu Province, Lanzhou University, Lanzhou, Gansu, China; 6 Academician Workstation, The Second Hospital of Lanzhou University, Lanzhou, Gansu, China

**Keywords:** atypical fibrous histiocytoma, literature review, pathological diagnosis, skull tumor, temporal bone

## Abstract

**Objective:**

Atypical fibrous histiocytoma (AFH) is an uncommon intermediate-grade fibrohistiocytic tumor that typically arises in the dermis or superficial soft tissues. Primary involvement of the skull is exceedingly rare and poses significant diagnostic challenges. We report a rare case of primary AFH arising in the temporal bone of an adult patient and provide a focused review of previously reported skull-based cases to clarify its clinicopathologic features, diagnostic pitfalls, and management considerations.

**Methods:**

A 34-year-old man presented with progressive right-sided tinnitus and sensorineural hearing loss. Computed tomography and magnetic resonance imaging demonstrated an osteolytic temporal bone lesion with intracranial extension and compression of the adjacent temporal lobe. Gross total surgical resection was performed. Detailed histopathological evaluation and an extended immunohistochemical panel were used to establish the diagnosis. A literature review of reported skull AFH cases was conducted for comparison.

**Results:**

Histologic examination revealed a moderately cellular spindle-cell tumor arranged in fascicles and storiform patterns, accompanied by multinucleated giant cells, hemosiderin-laden macrophages, and reactive bone formation. Immunohistochemistry showed diffuse vimentin positivity, weak cytoplasmic CD68 expression, and a Ki-67 proliferation index of approximately 20%, with negative staining for epithelial, melanocytic, neural crest, smooth muscle, and Langerhans cell markers. These findings supported the diagnosis of atypical fibrous histiocytoma. Postoperatively, tinnitus improved, while hearing loss showed limited recovery. No evidence of recurrence was observed during follow-up.

**Conclusion:**

Primary AFH of the temporal bone is an exceptionally rare entity that can mimic other destructive skull lesions on imaging and intraoperative inspection. Accurate diagnosis relies on careful histopathologic evaluation and exclusion of histologic mimickers using a comprehensive immunohistochemical panel. Complete surgical excision remains the cornerstone of treatment, and long-term follow-up is recommended due to the tumor’s intermediate malignant potential.

## 1 Introduction

Atypical fibrous histiocytoma (AFH) is an uncommon mesenchymal neoplasm classified as an intermediate-grade fibrohistiocytic tumor (1). It most frequently arises in the dermis and superficial soft tissues of young to middle-aged adults (2). Although representing only a minor proportion of fibrohistiocytic lesions, cutaneous AFH is relatively well characterized and typically presents as a slow-growing nodular mass with low but definite potential for local recurrence (1). In contrast, primary involvement of deep soft tissue or bone is exceedingly rare, and AFH arising in the skull constitutes one of the least frequently documented presentations (3–5).

Osseous AFH, particularly within the calvarium or skull base, poses significant diagnostic difficulty (5–7). Clinical symptoms are nonspecific, and radiologic findings often overlap with those of more common osteolytic skull lesions, including giant cell tumor, Langerhans cell histiocytosis, eosinophilic granuloma, osteolytic meningioma, sarcoma, or metastatic disease (5, 7–9). Imaging typically demonstrates lytic bone destruction with variable soft-tissue extension, but these features lack diagnostic specificity, making histopathology and immunohistochemistry essential for accurate identification (5, 7). Classic microscopic features include spindle-cell proliferation, multinucleated giant cells, hemosiderin-laden macrophages, and variable atypia, accompanied by an immunophenotype generally positive for vimentin and variably reactive for CD68 (6, 7).

Only a small number of skull-based AFH cases have been reported, most involving pediatric or adolescent patients and only rarely occurring in adults (9, 10). Although gene rearrangements such as EWSR1 or FUS may be detected—more typically in angiomatoid fibrous histiocytoma—these alterations are not consistently present and are not required for diagnosis (11, 12). Owing to the scarcity of reported cases, the natural history and optimal management strategy for cranial AFH remain undefined, though complete surgical excision with adequate margins remains the primary determinant of recurrence risk (7, 10, 13).

Here, we present a rare case of primary AFH arising in the temporal bone of an adult male, manifesting with tinnitus and progressive sensorineural hearing loss. We detail the clinical course, imaging findings, surgical management, and histopathologic features of this lesion, and contextualize the case within the existing literature. This report highlights the importance of including AFH in the differential diagnosis of destructive skull lesions and underscores the crucial role of comprehensive pathological evaluation.

## 2 Case presentation

### 2.1 Patient information

A 34-year-old male presented with a one-month history of progressive right-sided tinnitus, a sensation of aural fullness, and intermittent temporal discomfort exacerbated by mastication. He denied head trauma, chronic otologic disease, infectious symptoms, weight loss, or systemic complaints. Neurological examination revealed reduced right-sided hearing acuity; all other cranial nerve, motor, sensory, and cerebellar assessments were unremarkable.

### 2.2 Imaging findings

**Figure 1:**
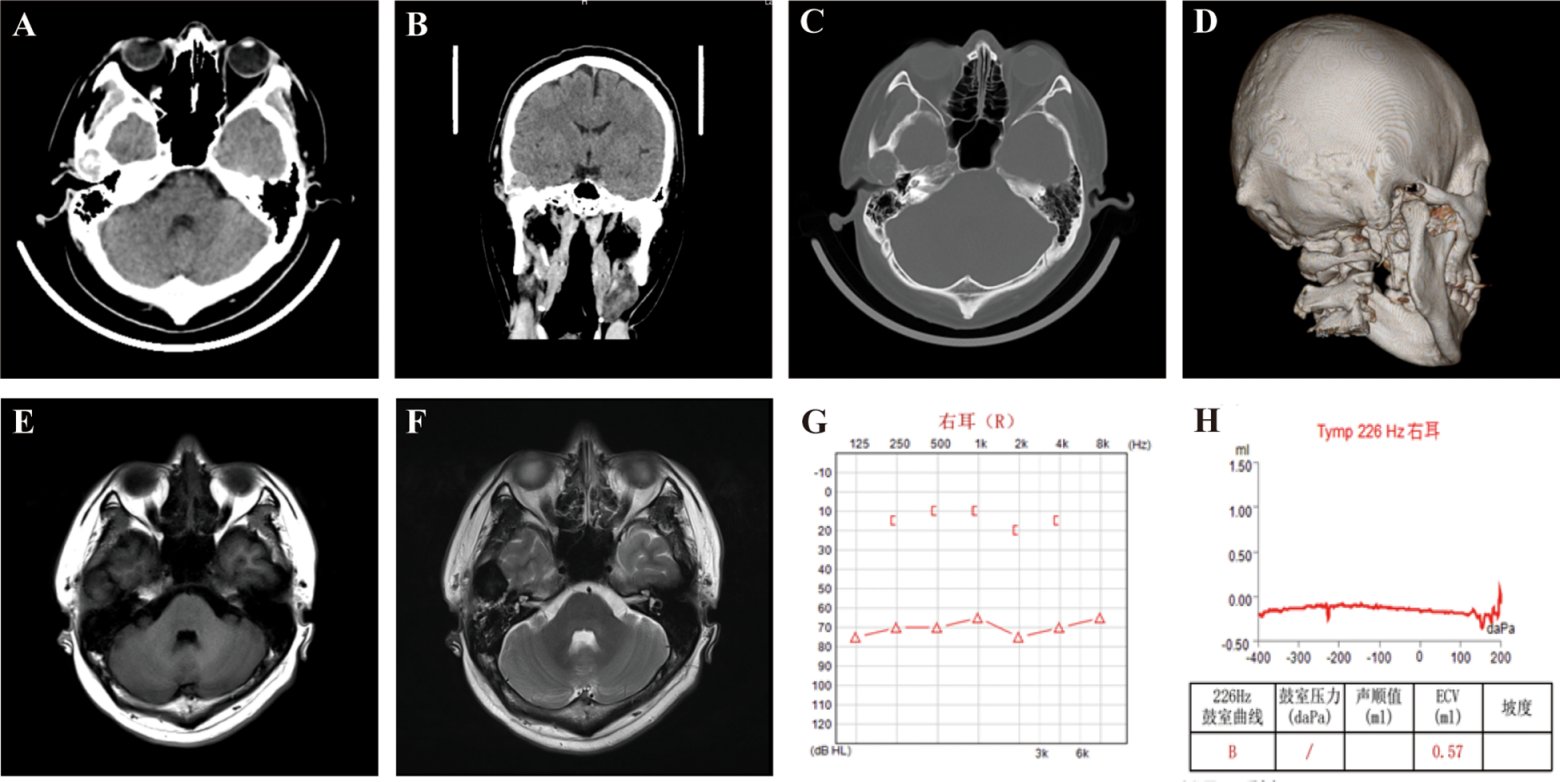
Preoperative imaging and audiological assessments of the patient demonstrating a right temporal bone mass and associated hearing impairment. **(A–D)** Axial and coronal CT **(A, B)**, bone window **(C)**, and 3D reconstruction **(D)** show osteolytic bone destruction in the right temporal bone with a soft tissue density mass, measuring approximately 17 × 14 mm. **(E, F)** MRI reveals a well-defined, lobulated lesion in the right temporal region, approximately 2.3 × 2.8 × 3.1 cm in size. The lesion shows iso- to slightly hypointense heterogeneous signals on T1-weighted imaging **(E)**, hypointense signals on T2-weighted imaging **(F)**. Adjacent brain parenchyma is compressed and displaced, and adjacent bone shows thinning and resorption. **(G)** Pure-tone audiometry indicating severe sensorineural hearing loss in the right ear across all tested frequencies. **(H)** Tympanometry of the right ear showing a Type B curve (flat), indicating middle ear effusion or dysfunction.

High-resolution temporal bone CT revealed a well-defined osteolytic lesion involving the right squamous temporal bone (17 × 14 mm), characterized by thinning and focal destruction of both the inner and outer tables. A soft-tissue component extended toward the middle cranial fossa, raising suspicion for an aggressive or infiltrative process (**Figures 1A–D**).

MRI demonstrated a lobulated extra-axial lesion measuring 2.3 × 2.8 × 3.1 cm, iso- to mildly hypointense on T1-weighted and predominantly hypointense on T2-weighted sequences, with heterogeneous post-contrast enhancement. Mild compression of the adjacent temporal lobe was observed, though no intraparenchymal invasion or diffusion restriction was present (**Figures 1E, F**).

Audiological testing revealed severe right-sided sensorineural hearing loss across all frequencies, a Type B tympanogram, and absent auditory brainstem response waveforms, indicating both conductive and neural pathway dysfunction likely attributable to mass effect on temporal bone structures (**Figures 1G, H**).

### 2.3 Surgical procedure

**Figure 2:**
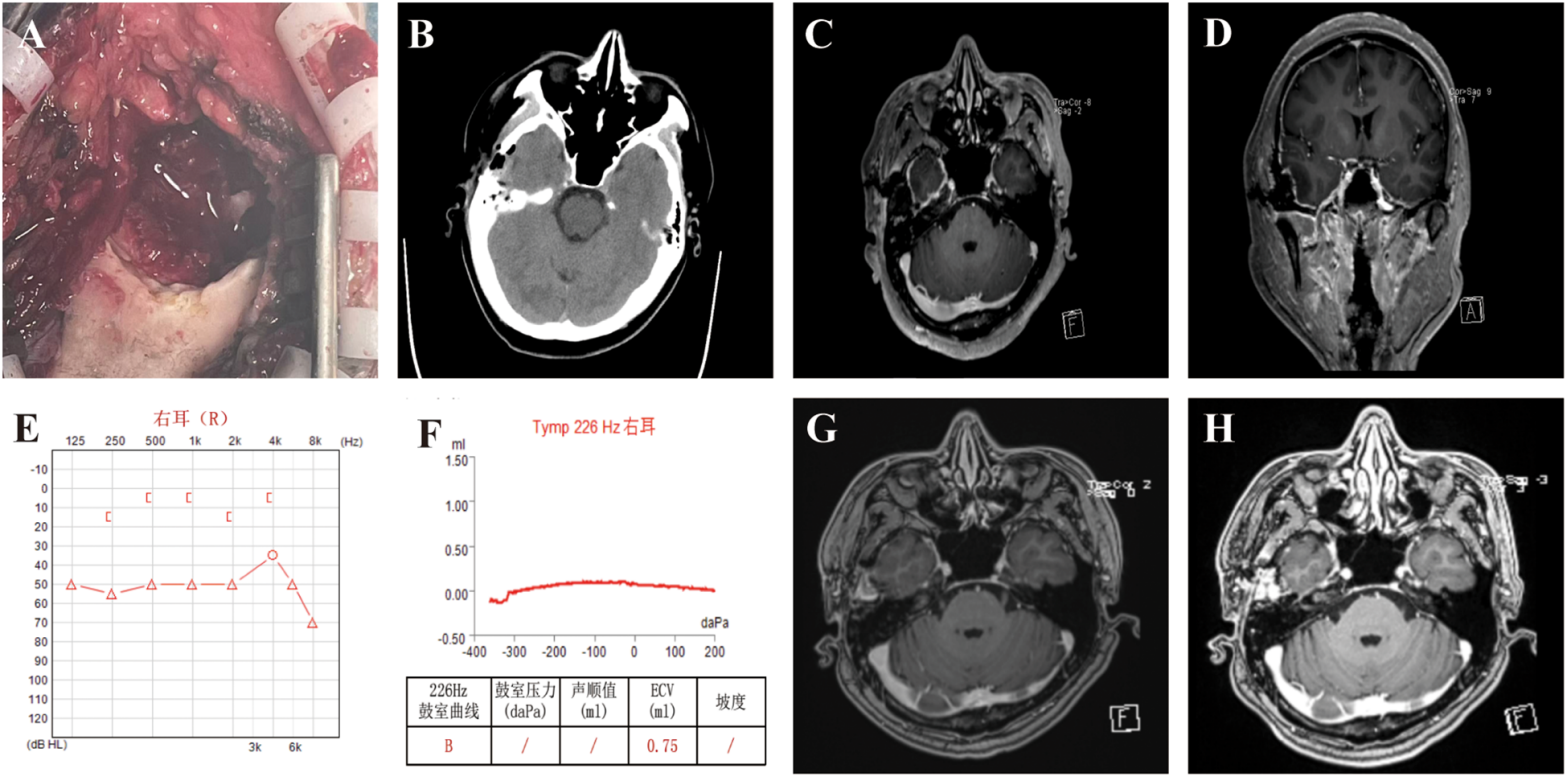
Composite illustration of intraoperative findings, postoperative imaging, and audiological assessments of the patient. **(A)** Intraoperative view showing bone destruction and tumor excision from the right temporal bone. **(B)** Postoperative non-contrast CT scan demonstrating no evidence of intracranial hemorrhage or acute complications. **(C, D)** Early postoperative brain MRI (axial and coronal T1-weighted images with contrast) showing no residual tumor in the right temporal bone region, with mild dural enhancement consistent with postoperative changes. **(E, F)** Postoperative audiological evaluation of the right ear. **(G, H)** Postoperative magnetic resonance imaging of the brain (axial T1-weighted imaging with contrast) at 1and 6 months postoperatively showed no signs of tumor recurrence.

The patient underwent a right temporal craniotomy. Intraoperatively, the mass appeared friable and brownish-red, with erosion through the inner skull table and focal infiltration of the dura (**Figure 2A**). The tumor lacked a true capsule and contained hemorrhagic and fibrous components. Gross total resection was achieved microsurgically. Skull margins were curetted to healthy bone, mastoid air cells were sealed with bone wax, and the dura was preserved without cerebrospinal fluid leakage. The postoperative course was uneventful.

### 2.4 Histopathological findings

**Figure 3:**
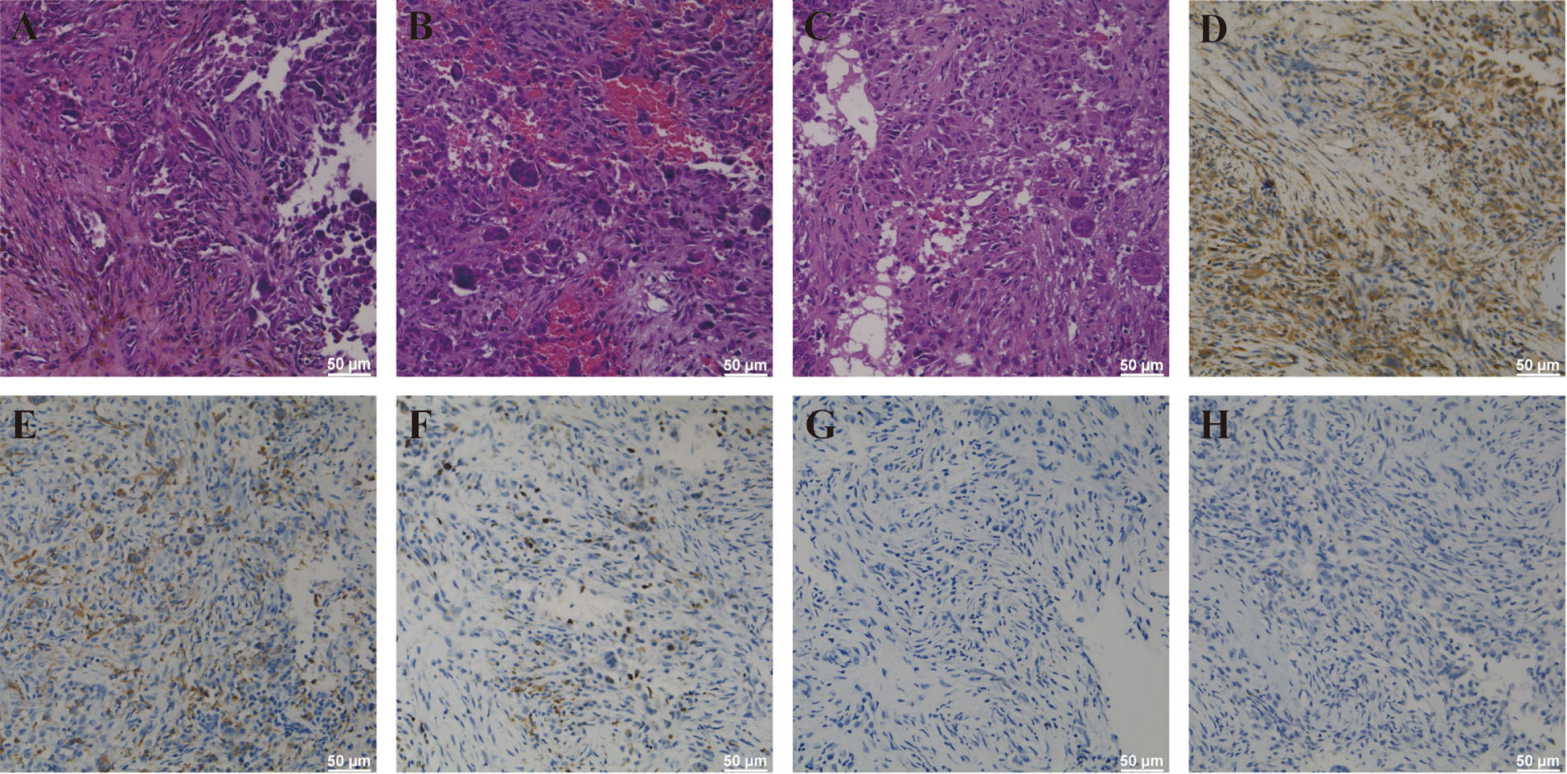
Pathological manifestations of AFH of the skull. **(A–C)** AFH HE staining showed that the cells were multinucleated giant cells, and some tumor cells were moderately heterotypic, with active growth and hemosiderin deposition. **(D)** The AFH cells are strongly positive with vimentin by immunohistochemistry. **(E)** Immunohistochemical staining showed CD68 expression for weakly positive in AFH cells. **(F)** Ki-67 immunostaining demonstrates a labeling index of approximately 20%. **(G)** Immunohistochemical staining showed that S-100 was negative in AFH cells. **(H)** Immunohistochemical staining showed that EMA was negative in AFH cells.

Microscopic evaluation revealed a moderately cellular spindle-cell neoplasm arranged in fascicles and storiform patterns, containing multinucleated giant cells, hemosiderin-laden macrophages, and areas of reactive bone formation. Mild to moderate cytologic atypia was noted, with no necrosis or atypical mitoses (**Figures 3A–C**).

Immunohistochemistry demonstrated diffuse vimentin positivity and weak CD68 expression, supporting fibrohistiocytic differentiation (**Figures 3D, E**). The tumor was negative for S100 (**Figure 3G**), SOX10, EMA (**Figure 3H**), cytokeratin, desmin, SMA, CD1a, CD163, HMB45, and Melan-A, excluding melanoma, meningioma, epithelial tumors, smooth muscle neoplasms, and Langerhans cell histiocytosis. The Ki-67 index was approximately 20%. Collectively, these findings supported the diagnosis of AFH (**Figure 3F**).

### 2.5 Postoperative follow-up

Postoperative CT and MRI confirmed complete excision with no residual lesion (**Figures 2B–D**). The patient’s tinnitus improved promptly, though audiologic reassessment showed only mild improvement in hearing thresholds, consistent with irreversible preoperative neural injury (**Figures 2E, F**). At one month and six months postoperatively, he remained clinically stable with no radiologic evidence of recurrence (**Figures 2G, H**).

## 3 Literature review

AFH predominantly arises in the dermis and superficial soft tissues, whereas primary involvement of bone, particularly the skull, remains exceedingly rare (7, 14, 15). To date, only a limited number of cranial or skull-based AFH cases have been reported, most of which are isolated case reports (**Table 1**). The earliest well-documented case of skull AFH was described by Black et al., involving an infant with a destructive calvarial lesion extending to the dura, who remained disease-free following complete surgical excision. This seminal report established the possibility of primary AFH arising within cranial bone and highlighted the importance of complete resection for favorable outcomes (5).

**Table 1:** Previously published skull/cranial AFH or related fibrous histiocytoma cases (summarized).

No.	Reference (year)	Age/Sex	Location (skull region)	Molecular testing	Treatment	Follow-up/Outcome
1	Black SPW et al. (5) (1980).	3-month-old infant (M)	Right parietal calvarium; bone destruction with dural invasion	Not reported	Surgical excision (gross total)	Long-term disease-free (reported~7 years)
2	Rao AJ et al. (16) (1986).	4-year-old girl (F)	Temporal bone/mastoid region	Not reported	Surgical excision	no recurrence reported at short-term follow-up
3	Granato L et al. (17), (2014).	Adult (age not specified)	Mastoid/temporal bone	Not reported	Surgical excision	Uneventful; pathology consistent with benign FH; follow-up uneventful (short-term).
4	Konstantinidis A et al. (10) (2019).	Pediatric	Intracranial AFH (various skull-related sites)	EWSR1 fusions reported in some cases	Surgery ± adjuvant management (case-dependent)	Variable; most patients without recurrence at published follow-up.
5	Sion AE et al. (18) (2020).	age reported	Intracranial location reported	Molecular testing variable in literature; case discusses angiomatoid FH features	Surgical excision	Favorable short-term outcome reported.
6	Gillon S et al. (9) (2021).	17-year-old girl (F)	Petrous/temporal bone with intracranial extension	EWSR1 rearrangement detected (reported)	Surgical excision	Reported as intracranial AFH; outcome favorable at reported follow-up.

Subsequent reports have described fibrous histiocytoma variants involving the skull, including both atypical and benign subtypes (7). Although benign fibrous histiocytoma (BFH) is histologically and biologically distinct from AFH, several skull-based BFH cases have been reported in the parietal, temporal, and skull-base regions (7, 19, 20). These cases are relevant in the differential diagnosis, as they share overlapping imaging features such as osteolytic bone destruction and soft-tissue extension, yet generally demonstrate less cytologic atypia and lower recurrence potential compared with AFH. Inclusion of these BFH cases in the literature provides a broader perspective on fibrohistiocytic tumors of the cranial skeleton.

In recent years, angiomatoid fibrous histiocytoma—a related but molecularly distinct entity—has also been reported in intracranial and skull-base locations, including the temporal bone. These cases frequently demonstrate EWSR1-related gene rearrangements and may present with hemorrhagic or cystic components (9, 12). While molecular alterations are not consistently identified in conventional AFH, these reports underscore the histologic and genetic heterogeneity within the fibrous histiocytoma spectrum and emphasize the need for careful morphologic and immunohistochemical correlation in cranial lesions (10, 21).

Radiologically, reported skull AFH cases consistently demonstrate nonspecific osteolytic changes with thinning or destruction of the inner and outer tables and variable epidural or extracranial soft-tissue components (9, 22). Such features often lead to preoperative misdiagnoses, including eosinophilic granuloma, giant cell tumor of bone, osteolytic meningioma, metastatic disease, or plasmacytoma (9, 22, 23). MRI findings are similarly variable, with lesions typically showing iso- to hypointense signals on T1-weighted images and heterogeneous enhancement after contrast administration (9, 22). These overlapping radiologic characteristics explain why AFH is rarely considered preoperatively in cranial lesions.

Histopathologically, previously published skull AFH cases show a consistent pattern of spindle-cell proliferation arranged in fascicles or storiform architecture, admixed with multinucleated giant cells, hemosiderin-laden macrophages, and variable degrees of cytologic atypia (7). Immunohistochemistry in most reports demonstrates vimentin positivity with variable CD68 staining, while melanocytic (S100/HMB45), epithelial (cytokeratin) and Langerhans-cell (CD1a) markers are usually negative (7, 24). Rearrangements involving EWSR1 (and only rarely FUS) are well documented in angiomatoid fibrous histiocytoma and are detectable in a substantial proportion of cases, but they are not universally present and their absence does not exclude the diagnosis, which therefore remains principally morphology- and IHC-based (11, 12).

Management trends across published cases emphasize the importance of achieving complete surgical excision, as recurrence is more likely after subtotal resection (7, 25). In available reports, patients who underwent gross total resection largely remained recurrence-free, whereas those with incomplete removal demonstrated higher risk of local progression (5, 7, 26). No clear role has been established for adjuvant radiotherapy or chemotherapy in conventional AFH of the skull.

In summary, the literature suggests that although AFH of the skull is rare, its clinicopathologic features are broadly consistent with AFH at other sites, while its radiologic presentation is frequently misleading. Thorough histopathologic and immunohistochemical evaluation remains essential for accurate diagnosis, and complete surgical excision remains the cornerstone of effective management.

## 4 Discussion

AFH represents an uncommon fibrohistiocytic neoplasm with intermediate malignant potential, most frequently arising in the dermis or superficial soft tissues (24, 27). Primary osseous involvement—and particularly localization in the skull—is exceptionally rare and introduces substantial diagnostic challenges for clinicians, radiologists, and pathologists (5, 7). The present case illustrates several clinically important aspects of skull-based AFH, including its nonspecific imaging appearance, histopathologic complexity, and management considerations (7, 9).

### 4.1 Diagnostic challenges and imaging differential diagnosis

In the skull, AFH often presents as an osteolytic lesion with soft tissue extension, a pattern shared by a broad spectrum of benign and malignant conditions (7, 22, 28). In our patient, CT and MRI demonstrated a destructive temporal bone mass resulting in compression of the adjacent temporal lobe. These features necessitated a wide differential diagnosis, including eosinophilic granuloma, giant cell tumor, osteolytic meningioma, Langerhans cell histiocytosis, plasmacytoma, metastatic disease, and low-grade sarcomas (22, 28). The heterogeneous enhancement pattern and absence of diffusion restriction suggested a non-high-grade lesion, yet imaging alone remained insufficient for narrowing the diagnosis (22, 29). These findings are consistent with previous reports, which emphasize that skull-based AFH rarely demonstrates imaging characteristics specific enough for preoperative identification (9, 30).

### 4.2 Intraoperative assessment and differential considerations

The intraoperative appearance of AFH often mirrors that of other fibrohistiocytic or osteolytic processes (31). In our case, the friable, brownish-red mass lacking a true capsule was highly suggestive of diagnoses such as giant cell tumor, aneurysmal bone cyst–like lesions, brown tumor, or even an aggressive fibrous lesion. The presence of focal hemorrhage and bone destruction further clouded intraoperative differentiation. As a result, surgeons must rely heavily on postoperative histopathology for definitive diagnosis, highlighting the importance of thorough sampling and communication between surgical and pathology teams (31–33).

### 4.3 Histopathologic features and diagnostic confirmation

AFH in bone exhibits considerable histologic overlap with other entities, making diagnosis primarily one of exclusion (7, 34). In our case, hallmark features—including spindle-cell fascicles, multinucleated giant cells, hemosiderin deposition, and foci of reactive bone formation—raised suspicion for AFH but required careful differentiation from histologic mimics (7). Pleomorphic sarcoma and osteosarcoma were excluded due to the absence of significant nuclear atypia, atypical mitotic figures, or malignant osteoid (7, 34). Langerhans cell histiocytosis was excluded based on negative staining for CD1a and absence of grooved nuclei (34). Melanoma and meningioma were ruled out through negativity for S100, SOX10, HMB45, EMA, and cytokeratin markers (34). Weak positivity for CD68 and strong expression of vimentin supported a fibrohistiocytic lineage (7, 34).

Although EWSR1 rearrangements are occasionally identified in angiomatoid fibrous histiocytoma, molecular testing is not required for the diagnosis of AFH and is not consistently reported in skull lesions (12, 35). In the present case, the combination of classic morphology, a supportive immunophenotypic profile, and exclusion of competing diagnoses provided robust confirmation of AFH despite the lack of molecular assays (7, 34). This aligns with prior literature indicating that AFH can be reliably diagnosed through conventional pathology when characteristic features are present (12, 34).

### 4.4 Pathogenesis and possible cell of origin

The underlying pathogenesis of AFH remains poorly understood (36). Proposed origins include fibroblasts, myofibroblasts, or histiocyte-lineage cells capable of variable mesenchymal differentiation (37, 38). The presence of hemosiderin-laden macrophages and multinucleated giant cells suggests an inflammatory or reparative component (9, 39). Some authors have hypothesized that chronic trauma, inflammation, or repeated local irritation may trigger aberrant fibroblastic proliferation, particularly in osseous sites (40). In the temporal bone, AFH may arise from mesenchymal precursor cells in the diploë or periosteum, although definitive evidence remains lacking due to the rarity of reported cases (9, 41).

### 4.5 Management considerations and prognosis

Consistent with prior reports, complete surgical excision remains the cornerstone of AFH management (42, 43). Local recurrence is strongly associated with incomplete resection, with reported rates ranging between 10% and 25% (26, 44–46). Skull lesions, because of limited anatomic margins and proximity to neurovascular structures, may pose additional challenges for achieving complete excision (9, 46). In our case, gross total resection was successfully achieved, and early follow-up showed no evidence of recurrence.

The role of adjuvant radiotherapy or chemotherapy in AFH remains unclear and is not routinely recommended unless malignant transformation or unresectable disease is present (43, 47). Given its intermediate malignant potential, long-term surveillance is advisable even after complete removal (42). Follow-up strategies typically include periodic imaging at 6–12 month intervals during the first several years (46).

### 4.6 Clinical significance of the present case

This case highlights several unique aspects that expand current understanding of cranial AFH. First, the adult age of presentation contrasts with many previously reported cases occurring in children or adolescents (7, 9, 18). Second, involvement of the temporal bone with associated sensorineural hearing loss is uncommon and adds a functional dimension rarely described in AFH literature (9). Third, the combination of destructive bony involvement, intraoperative infiltration of the dura, and a relatively elevated Ki-67 index underscores the biological variability of AFH and its potential for locally aggressive behavior despite its low metastatic risk (5, 7, 36, 45, 48, 49).

## 5 Conclusion

AFH of the skull is an exceptionally rare entity that poses significant diagnostic challenges due to its nonspecific clinical presentation, variable radiologic appearance, and broad histopathologic differential diagnosis. This case underscores the importance of considering AFH in the evaluation of destructive calvarial lesions, particularly when imaging findings are inconclusive and the intraoperative appearance mimics other fibro-osseous or neoplastic processes. Accurate diagnosis requires careful integration of morphology, immunohistochemistry, and exclusion of more aggressive mimickers.

Complete surgical excision remains the most effective treatment strategy and is critical for minimizing the risk of recurrence. Although the biological behavior of cranial AFH appears largely consistent with its soft-tissue counterparts, long-term follow-up is warranted due to its intermediate malignant potential. This case contributes to the limited body of literature by documenting an uncommon temporal bone presentation with associated auditory dysfunction, highlighting the need for increased awareness of this rare diagnosis among neurosurgeons, pathologists, and otologic specialists.

## Data Availability

The original contributions presented in the study are included in the article/supplementary material. Further inquiries can be directed to the corresponding authors.
